# Resting state functional connectivity in the human spinal cord

**DOI:** 10.7554/eLife.02812

**Published:** 2014-08-05

**Authors:** Robert L Barry, Seth A Smith, Adrienne N Dula, John C Gore

**Affiliations:** 1 Vanderbilt University Institute of Imaging Science, Nashville, United States; 2 Department of Radiology and Radiological Sciences, Vanderbilt University Medical Center, Nashville, United States; 3 Department of Biomedical Engineering, Vanderbilt University, Nashville, United States; Oxford University, United Kingdom

**Keywords:** fMRI, spinal cord, 7 Tesla, resting state, functional connectivity, human

## Abstract

Functional magnetic resonance imaging using blood oxygenation level dependent (BOLD) contrast is well established as one of the most powerful methods for mapping human brain function. Numerous studies have measured how low-frequency BOLD signal fluctuations from the brain are correlated between voxels in a resting state, and have exploited these signals to infer functional connectivity within specific neural circuits. However, to date there have been no previous substantiated reports of resting state correlations in the spinal cord. In a cohort of healthy volunteers, we observed robust functional connectivity between left and right ventral (motor) horns, and between left and right dorsal (sensory) horns. Our results demonstrate that low-frequency BOLD fluctuations are inherent in the spinal cord as well as the brain, and by analogy to cortical circuits, we hypothesize that these correlations may offer insight into the execution and maintenance of sensory and motor functions both locally and within the cerebrum.

**DOI:** http://dx.doi.org/10.7554/eLife.02812.001

## eLife digest

Brain imaging methods such as functional magnetic resonance imaging (fMRI) can provide us with a picture of what the brain is doing when a person is carrying out a specific task. For example, an fMRI scan recorded whilst someone is reading is likely to show activity in regions in the left hemisphere of the brain that are known to be involved in language comprehension. fMRI can also be used to measure patterns of neuronal activity when someone is awake but not engaged in a specific task. This approach, known as resting state fMRI, can be used to examine which regions of the resting brain are active at the same time. Researchers are interested in these patterns of brain activity because they reflect neural circuits that work together to produce different functions and behaviors.

Over 4000 papers have used resting state fMRI to study the human brain. However, to date there has been no conclusive investigation of resting state activity in the spinal cord. This is largely because the spinal cord is much smaller than the brain, and most fMRI scanners are not sensitive enough to study it in detail. Consequently, little is known about intrinsic neural circuits in the resting spinal cord.

Now Barry et al. have used advances in fMRI technology to show that resting state functional connectivity does indeed exist in the spinal cord. Correlations were found in the resting levels of activity between spatially distinct areas of the cord, specifically between the ventral horns and between the dorsal horns. The ventral horns relay motor signals to the body, whilst the dorsal horns receive sensory signals from the body.

These findings also have clinical applications. Some patients with incomplete spinal cord injuries can recover near normal function, but the mechanisms responsible for this recovery are unclear because clinicians have not been able to probe neuronal connections in the spinal cord in a non-invasive manner. The work of Barry et al. should help with efforts to understand the neuronal changes that support recovery from spinal cord injury.

**DOI:** http://dx.doi.org/10.7554/eLife.02812.002

## Introduction

Since the early 1990s, thousands of functional magnetic resonance imaging (fMRI) studies have offered new insights into the functional architecture of the brain and have significantly increased our understanding of normal and aberrant brain functions. The earliest papers investigated task-based fMRI, where evoked BOLD signal changes were interpreted as hemodynamic responses subsequent to neural activity (Ogawa et al., 1990; Bandettini et al., 1992; Kwong et al., 1992; Ogawa et al., 1992) and were used to infer which brain regions were activated for a specific task. The range and impact of fMRI methods were expanded in 1995 when Biswal et al. established the existence of correlations between low-frequency (< 0.08 Hz) BOLD signals from spatially distinct locations when no task was performed (Biswal et al., 1995), and over 4000 subsequent papers have documented different aspects of resting state fMRI. Importantly, these correlations have been widely adopted to infer functional connectivity between cortical regions (Greicius et al., 2003; Fox et al., 2005; Smith, 2012). The identification of patterns of highly correlated low-frequency signals in the resting brain provides a powerful approach to delineate and describe neural circuits, and an unprecedented insight into the manner in which distributed regions work together to achieve specific functions (Pizoli et al., 2011). In this study, we present the first robust demonstrations that similar phenomena can be detected within the gray matter of the human spinal cord, and we report our preliminary attempts to perform resting state connectivity studies within the cords of normal volunteers.

Although the vast majority of fMRI studies have explored function in the cerebrum, there have been a few investigations of function in the human brainstem and spinal cord. fMRI in the spinal cord was first performed (Yoshizawa et al., 1996) in 1996, and task-based (motor and/or sensory) spinal fMRI has since been demonstrated by a handful of groups worldwide (Stroman et al., 1999; Backes et al., 2001; Madi et al., 2001; Giove et al., 2004; Moffitt et al., 2005; Maieron et al., 2007; Giulietti et al., 2008; Agosta et al., 2009a; Cohen-Adad et al., 2010; Summers et al., 2010; Brooks et al., 2012; Sprenger et al., 2012). Spinal cord fMRI has primarily been used to study motor and sensory/pain pathways in the healthy spinal cord, but has also been shown to be sensitive to changes in patients with spinal cord injury (Stroman et al., 2002, 2004; Kornelsen and Stroman, 2007) and multiple sclerosis (Agosta et al., 2008a, 2008b, 2009b; Valsasina et al., 2010, 2012). Importantly, these spinal fMRI studies were focused on understanding spinal cord function when performing tasks, and to date only one paper has reported an investigation of resting state BOLD fluctuations in the human spinal cord, from which the results were equivocal (Wei et al., 2010). Partly, the lack of positive reports may reflect the relatively poor signal-to-noise ratio of spinal cord images achievable at conventional field strengths (1.5 Tesla and 3.0 Tesla) and the inherent limitations of low spatial resolution in studying small structures. The advent of ultra-high magnetic fields (7 Tesla and above) and implementation of appropriate multichannel spinal cord coils, along with improved image acquisition and correction protocols, provides new opportunities for high-resolution fMRI of the spinal cord with increased sensitivity to BOLD fluctuations in the small gray matter structures that are typically not well visualized at lower fields.

**Figure 1.**
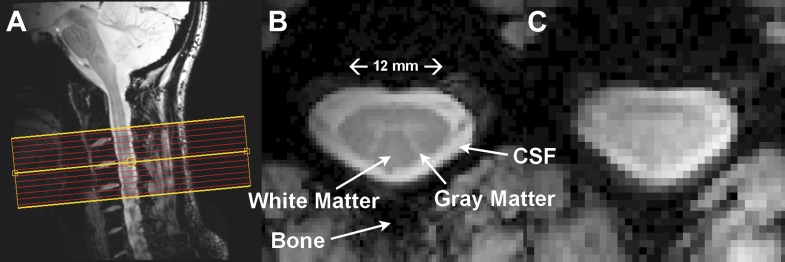
Resting state spinal cord fMRI at 7 Tesla. (**A**) Mid-sagittal slice from a healthy volunteer showing the complete cervical cord and typical axial slice placement for this resting state study. In all subjects the imaging stack was centered on the C3/C4 junction, providing full coverage of C3 and C4 and partial coverage of C2 and C5. (**B**) T_2_^*^-weighted anatomical image at C4 acquired with 0.6 × 0.6 × 4 mm^3^ voxels and interpolated to 0.31 × 0.31 × 4 mm^3^. Excellent contrast permits visualization of the characteristic butterfly-shaped gray matter column. (**C**) A single T_2_^*^-weighted functional image of this axial slice (acquired with 0.91 × 0.91 × 4 mm^3^ voxels). Functional images are high quality with minimal geometric distortions and T_2_^*^ blurring and permit adequate spatial delineation between white matter and cerebrospinal fluid. **DOI:** http://dx.doi.org/10.7554/eLife.02812.003

The spinal cord is essentially a long, cylindrical neural structure responsible for relaying motor and sensory information between the brain and body, it sits within a bath of cerebrospinal fluid (CSF), and is surrounded by large vertebral bodies and intervertebral discs (Figure 1A). A butterfly-shaped gray matter structure surrounded by densely packed white matter is found within the cord (Figure 1B). The gray matter is anatomically described by ventral (anterior), lateral, and dorsal (posterior) horns, though the lateral and ventral horns in thoracic and lumbar segments are often summarized as anterior (ventrolateral) gray matter. The dorsal horn contains neurons that receive sensory information from the extremities while upper motor neurons synapse onto lower motor neurons in the ventral horn, which relay information to the extremities (Kandel et al., 2000).

The relatively small number of spinal fMRI studies to date and lack of well-developed investigative tools make it difficult to formulate clear hypotheses of what low-frequency (< 0.08 Hz) BOLD signal correlations may be expected between sub-regions of spinal cord gray matter in a resting state. However, the formulation of such hypotheses may, in part, be guided by known anatomical connections or spinal cord function. For example, the likely existence of central pattern generators in the human spinal cord that subserve basic locomotion (Kandel et al., 2000, p. 753) suggest that functional connectivity may exist between ventral (motor) horns. Similarly, reflexes suggest connections between a dorsal (sensory) horn and both ipsilateral and contralateral ventral horns (Kandel et al., 2000, Figure 36-2), and are primarily apparent in the presence of noxious stimuli. The ascending sensory pathways and descending motor pathways also suggest that there may be connectivity along the length of the cord, at least within individual dermatomes for dorsal horns (Kandel et al., 2000, p. 445). However, it must be emphasized that the lack of a direct anatomical connection between two sub-regions of spinal gray matter does not preclude the possibility of finding connectivity between these regions because they may be indirectly connected via other pathways. In practice, even if significant low-frequency signal variations related to function are manifest, they may be obscured by cord motion and various other sources of physiological noise. Here we adapt the paradigm used for earlier investigations of connectivity in the brain whereby we define very small regions of interest in anatomically distinct parts of spinal gray matter which in general subserve defined functions, and examine interregional steady-state correlations between them. In addition, we derive the patterns of voxels that show significant temporal correlation with selected single voxels within regions. These approaches have been successfully used, for example, in the cortex to delineate motor circuits. The use of high resolution images at 7 Tesla permit the reliable separation of ventral, dorsal, and bilateral segments of the cord so we can examine functional connectivity between sub-regions guided by known anatomical features. Functional connectivity along the cord may also be examined by considering any subregion in one slice and the same or another subregion in adjacent (or other) slices, but here we limit the group analysis of our first report to functional connectivity assessed within axial slices.

## Results

**Figure 2.**
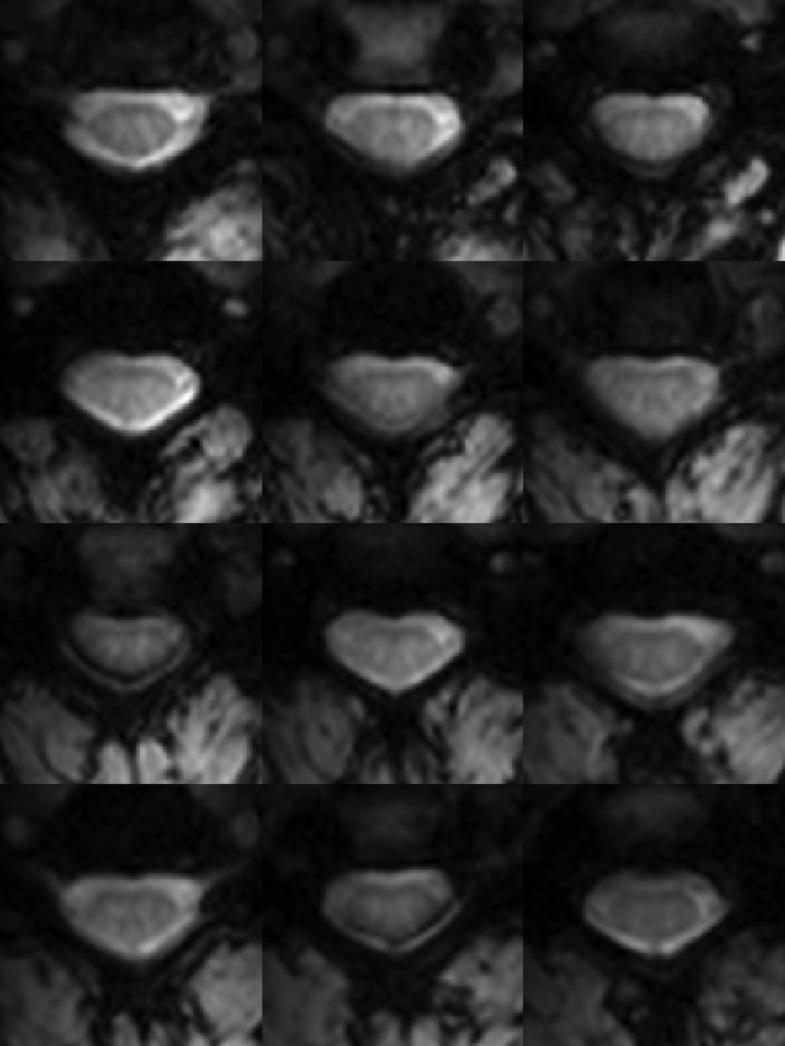
Functional weighted spinal cord images at 7 Tesla. A single volume of twelve contiguous T_2_*-weighted slices centered on the C3/C4 junction (as illustrated in Figure 1A) in one subject. Each volume was acquired with 0.91 × 0.91 × 4 mm^3^ voxels and resampled to 0.31 × 0.31 × 4 mm^3^ voxels during the affine functional-to-anatomical registration. Excellent contrast between white matter and cerebrospinal fluid facilitates accurate registration between such functional volumes and high-resolution anatomical images (Figure 1B). The use of a 3D acquisition sequence with relatively short echo time and relatively few k-space lines per radiofrequency pulse provides high-quality images with minimal signal drop-out and geometric distortions, although artifacts caused by fat shift of the nerve root sleeve in the phase-encode direction still affect the dorsal edge in a few slices. **DOI:** http://dx.doi.org/10.7554/eLife.02812.004

**Figure 3.**
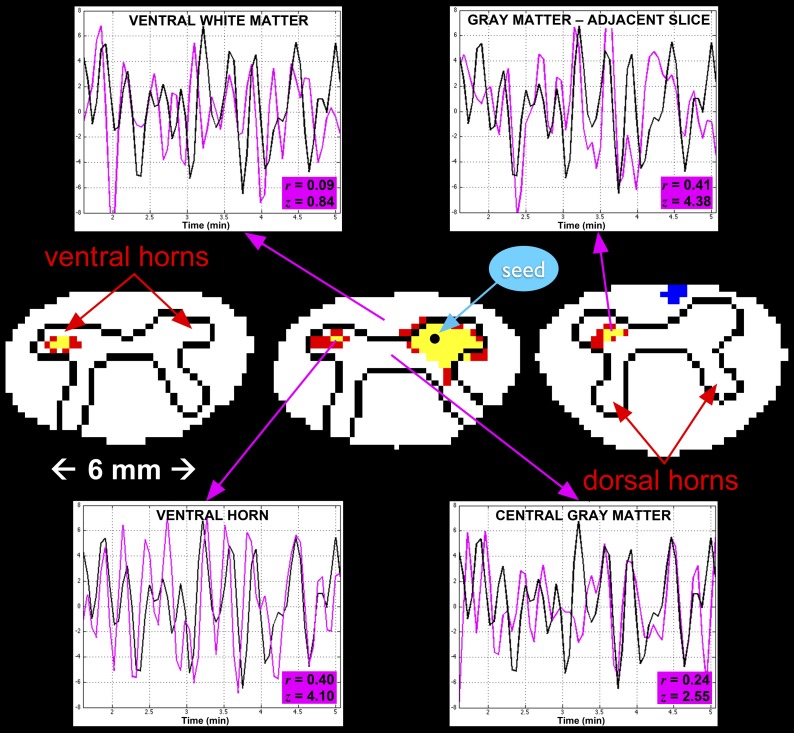
A single-subject analysis of resting state functional connectivity with corresponding time series. For clarity, only outlines of the gray matter butterfly and surrounding white matter are shown (rostro-caudal from left to right). Red and yellow represent statistically significant positive correlation with the seed time series (using a two-sided 99.9% confidence interval where red is 3.29 < *z* ≤ 3.89 and yellow is *z* > 3.89), and blue represents negative correlation (*z* < −3.29). The seed voxel is selected in the right ventral horn in C5, and exhibits functional connectivity with the contralateral ventral horn in the same slice as well as adjacent slices. Such connectivity between ventral horns is observed across all subjects. In each of the four plots, a 3.5-min segment of the seed time course is shown in black and the time course of the corresponding region of interest is shown in magenta. The highest correlations are observed in the contralateral ventral horn on the same slice (*z* = 4.10) and on the adjacent slices (*z* = 4.38). Correlations with central gray matter (*z* = 2.55) and adjacent white matter (*z* = 0.84) are relatively low, which, given the small size of the spinal cord, suggest that such correlations are genuine and not dominated by widespread physiological noise. **DOI:** http://dx.doi.org/10.7554/eLife.02812.005

**Figure 4.**
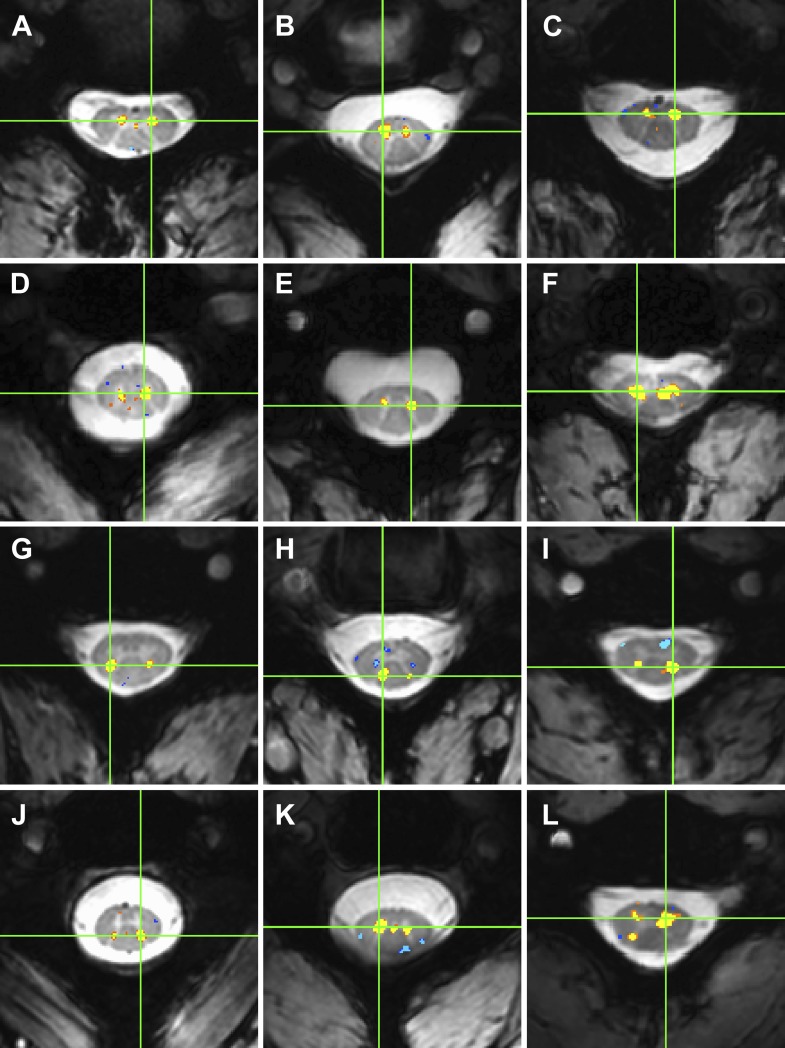
Examples of within-slice resting state functional connectivity across subjects. These analyses were performed using AFNI's ‘InstaCorr’ with p < 0.001 and no minimum cluster size. In each panel, a seed voxel is marked with a green crosshair and resultant correlations are overlaid on the anatomical image. (**A**)–(**F**) Connectivity between ventral horns for subjects 1, 3, 8, 10, 11, and 13, respectively. (**G**)–(**J**) Connectivity between dorsal horns for subjects 5, 16, 18, and 22, respectively. (**K** and **L**) Less common correlations within gray matter. In (**K**) (subject 20), focal connectivity between ventral horns and with central gray matter. In (**L**) (subject 7), connectivity between ventral horns but also with the contralateral dorsal horn. At the single-subject level, there is some evidence for functional connectivity between ventral and dorsal horns, but such correlations are less common across slices and not statistically significant at the group level. **DOI:** http://dx.doi.org/10.7554/eLife.02812.006

Functional images were preprocessed to mitigate rigid-body motion and physiological noise, and spatially interpolated to match the digital resolution of the T_2_^*^-weighted anatomical images (Figure 2). A 14-step standardized analysis protocol (described in 'Materials and methods') was used for each of the 22 subjects studied. In each subject, temporal signal-to-noise ratio (TSNR) was measured in spinal gray matter upon completion of the functional-to-anatomical affine registration (step #9) as well as after the application of CSF and white matter ‘regressors of no interest’ (steps #11 and #12). Across all 22 subjects, we observed a 30% increase in median TSNR (from 29.3 to 38.1) after the application of these few regressors, demonstrating the importance of characterizing and removing structured noise sources (Xie et al., 2012). After band-pass filtering to isolate the frequency range of interest (0.01–0.08 Hz), single-subject analyses show that statistically significant correlations are measurable between selected regions and are reproducible across subjects. As an illustrative example with the corresponding time series, an analysis performed on one subject (female, 23 years old) demonstrates connectivity with the contralateral ventral horns in the same slice and with adjacent slices when a seed region is selected in the center of the right ventral horn (Figure 3). A stringent threshold of |*z*| > 3.29 (a two-tailed 99.9% confidence interval) was selected to show that connectivity is focused in the gray matter horns and not in central gray matter (connecting left and right sides and largely dominated by the central canal) nor adjacent white matter, which provides evidence that such gray matter correlations cannot be simply attributed to spatially correlated physiological noise and more likely represent genuine functional connectivity. Further examples of within-slice connectivity analyses in single subjects confirm that reproducible focal connectivity is found between ventral horns (Figure 4A–F) and between dorsal horns (Figure 4G–J). There is also evidence of plausible connectivity with central gray matter (Figure 4K) and between ventral and dorsal horns (Figure 4L), but these correlations are less consistent across all slices and not statistically significant at the group level. To quantify the occurrence of within-slice correlations between gray matter sub-regions across slices, we averaged time courses within each respective gray matter sub-region (defined in step #14) and considered only positive correlations at a more conventional 95% confidence interval (*z* > 1.65; one-tailed). Across all 264 slices (12 slices/subject × 22 subjects), we observed that 67% of slices (177 of 264) exhibit significant correlations between ventral horns and 37% of slices (97 of 264) exhibit significant correlations between dorsal horns. In comparison, a markedly fewer number of slices (only 1 in 5) exhibited significant correlations between the remaining four pairs: 21% between left ventral and left dorsal horns (55 of 264), 21% between left ventral and right dorsal horns (55 of 264), 20% between right ventral and left dorsal horns (54 of 264), and 23% between right ventral and right dorsal horns (62 of 264).

**Figure 5.**
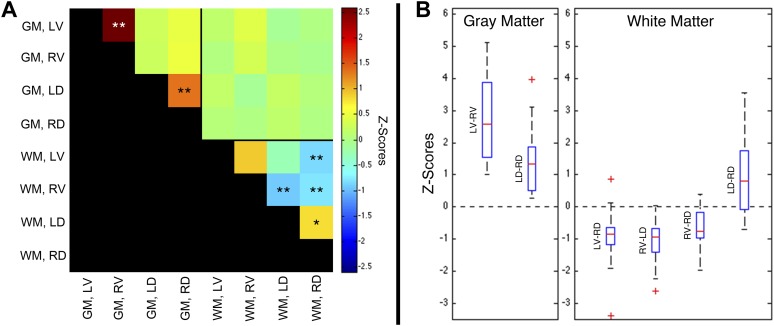
Group-level functional connectivity between sub-regions of spinal gray matter (GM) and surrounding white matter (WM) within slices. (**A**) In GM, strong positive correlations are observed between left (LV) and right (RV) ventral horns, as well as left (LD) and right (RD) dorsal horns (*p<0.05; **p<0.01; Bonferroni corrected). Weaker positive and negative correlations are observed within WM. No statistically significant correlations are observed between spinal GM and WM (upper right quadrant). (**B**) Box-and-whisker plots showing the median and upper and lower quartiles of the six statistically significant results identified in (**A**). Whiskers extend out to 1.5 times the distance between the upper and lower quartiles, and an outlier (beyond the whiskers) is denoted by a plus. Wilcoxon signed rank tests identify the distributions of *z*-scores (across all slices and subjects) that are significantly different from zero (p < 0.05). Functional connectivity between WM sub-regions is more variable and exhibits both positive and negative median correlations. In comparison, median GM correlations between LV-RV and LD-RD are positive across all 22 subjects. Additional analysis permutations (described in Figure 5—figure supplements 1, 3, and 5 with example GM power spectra shown in Figure 5—figure supplements 2, 4, and 6) reveal that the three negative WM correlations are influenced by WM regression (step #12) and thus are open to more than one interpretation. However, supplementary analyses reveal that the positive GM correlations between ventral horns and between dorsal horns persist across all preprocessing permutations. These additional analyses further support the conclusion that positive correlations between GM horns are not artifactual—possibly created by preprocessing choices or frequency bandwidth selection—and most likely represent genuine functional connectivity. **DOI:** http://dx.doi.org/10.7554/eLife.02812.007 10.7554/eLife.02812.008Figure 5—source data 1.Matlab file containing the raw data used to generate Figure 5.The dimensions of this matrix are 8 × 8 × 22 where the 8 × 8 represents all possible comparisons between the eight sub-regions (Figure 5A) and the upper-triangular entries are non-zero. For each of the 28 unique comparisons, the 22 entries in the third dimension correspond to the median measurement of functional connectivity across all 12 slices for each of the 22 subjects.**DOI:** http://dx.doi.org/10.7554/eLife.02812.008

A group-level analysis of functional connectivity between sub-regions of spinal cord gray matter and adjacent white matter confirmed that the most robust correlations are observed between left and right ventral (motor) horns (p < 0.01; corrected), as well as between left and right dorsal (sensory) horns (p < 0.01; corrected) (Figure 5A). No significant group-level correlations (p > 0.05) were observed between other gray matter sub-regions, nor between spinal cord gray and white matter. Weak positive correlations were observed between left and right dorsal column white matter (p < 0.05; corrected), and negative correlations were observed between left ventral white matter and right dorsal white matter (p < 0.01; corrected), and between right ventral white matter and both left (p < 0.01; corrected) and right (p < 0.01; corrected) dorsal white matter. The apparent existence of negative correlations in resting state spinal cord data is not unexpected because anticorrelations are commonly observed in resting state analyses of the brain (Chang and Glover, 2010) and have been a topic of intense discussion for over a decade. The ranges of values within these six statistically significant distributions are presented as box-and-whisker plots (Figure 5B). The lower quartile is above zero in both gray matter plots, demonstrating that positive gray matter connectivity is a robust and reproducible measurement. In comparison, temporal correlations between white matter sub-regions are more variable and exhibit both positive and negative median correlations. The raw data used to generate this figure is provided as Figure 5—source data 1. Additional analyses were performed (Figure 5—figure supplement 1, Figure 5—figure supplement 3, and Figure 5—figure supplement 5) to confirm that positive gray matter correlations are stable across various preprocessing procedures whereas white matter correlations are positive before but negative or non-significant after white matter regression (step #12). These supplementary analyses also showed that weaker positive correlations between sub-regions of left and right dorsal white matter remained positive and significant across various preprocessing configurations.

**Figure 5—figure supplement 1.**
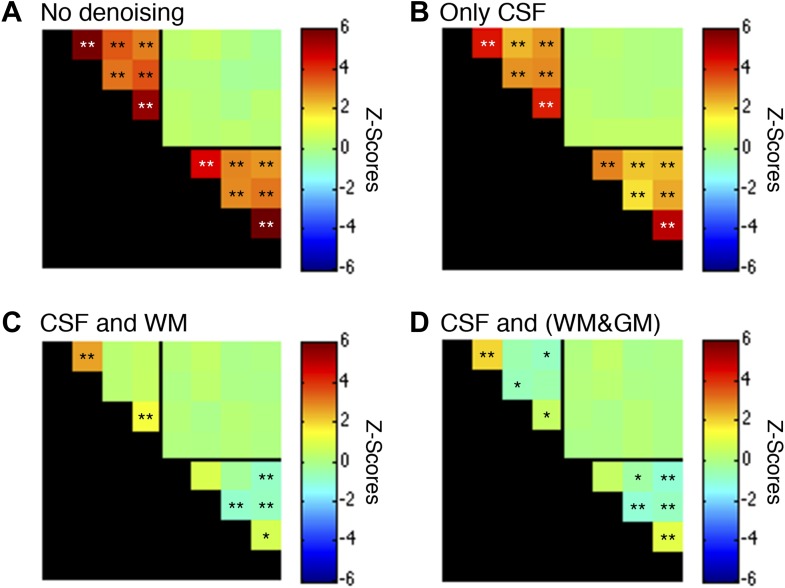
Functional connectivity matrices resulting from preprocessing pipeline permutations. As before, functional data were band-pass filtered between 0.01 and 0.08 Hz (*p<0.05; **p<0.01; Bonferroni corrected). For clarity the labels are not shown for each column/row but are the same as in Figure 5. (**A**) Preprocessing was performed as described in the Methods except CSF and WM regressors (steps #11 and #12) were not applied. Each GM sub-region is highly correlated with all other GM sub-regions and similarly each WM sub-region is highly correlated with all other WM sub-regions. Interestingly, there are no significant group-level correlations between GM and WM sub-regions, suggesting that ‘global’ GM fluctuations tend to be constrained to GM and ‘global’ WM fluctuations tend to be constrained to WM. (**B**) Preprocessing was performed as described in ‘Materials and methods’ except a WM regressor (step #12) was not applied. The application of only CSF regressors reduced correlations within both GM and WM, but all inter-region correlations remained and were statistically significant. (**C**) Preprocessing was performed exactly as described in ‘Materials and methods’ (i.e., this panel is the same as Figure 5A except for a larger dynamic range for *z*-scores). Regression of the principal eigenvector of all time series in the WM mask significantly altered correlations within both GM and WM. This unintuitive result may be explained if WM masks contain signal contributions from adjacent GM voxels, which is certainly possible given the small size of the GM butterfly and unavoidable partial volume effects, and sub-millimeter functional-to-anatomical warping inaccuracies caused by magnetic field inhomogeneities. (**D**) To investigate the possibility that WM masks contain fractions of GM voxels, and the impact of different regression masks on correlations between sub-regions, preprocessing was performed as described in ‘Materials and methods’ except step #12 extracted the principal eigenvector of all time series within a combined WM and GM mask. Given the small size of each GM mask relative to its neighboring WM mask, the principal eigenvector from the combined mask (WM&GM) should be similar to only using WM. Indeed, regression with these modified eigenvectors produces similar group-level correlations between WM sub-regions and introduce weak negative correlations between GM sub-regions. In GM, positive correlations between ventral horns and between dorsal horns still persist after this more invasive regressor, providing further evidence that these strong temporally correlated fluctuations are unlikely to be caused by physiological motion or spatially-correlated noise. To further investigate the spatial extent of global fluctuations within GM, we repeated the preprocessing as described in ‘Materials and methods’ but eroded the WM mask slightly before extracting the principal eigenvector. The results of this tertiary analysis (not shown) were slightly different but statistically similar to Figure 5A, demonstrating that voxels on and near the GM-WM boundary in fact characterize physiological fluctuations from both tissue types due to unavoidable partial volume effects and sub-millimeter warping inaccuracies. Overall, these supplementary analyses suggest that the preprocessing performed in the main manuscript (regression of the principal eigenvector from WM masks without erosion) is an appropriate strategy for suppressing extraneous fluctuations within both WM and GM without introducing substantial negative correlations between GM sub-regions. Finally, power spectra for WM and GM sub-regions in (**C**) are presented in Figure 5—figure supplement 2. **DOI:** http://dx.doi.org/10.7554/eLife.02812.009

**Figure 5—figure supplement 2.**
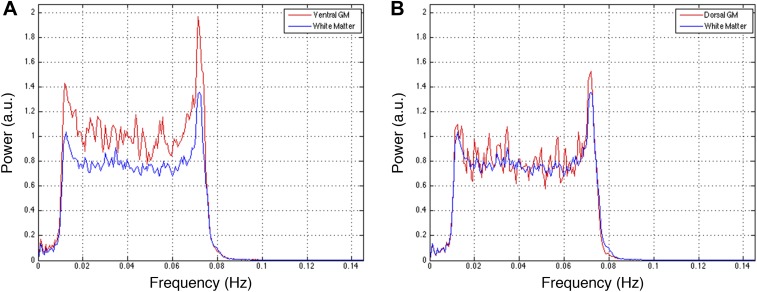
Power spectra across gray and white matter sub-regions for data filtered between 0.01 and 0.08 Hz. Power spectra for (**A**) ventral and (**B**) dorsal GM sub-regions in Figure 5—figure supplement 1C that exhibit significant positive correlations (*z* > 1.65; one-tailed), and all WM sub-regions. For each frequency the plotted power represents median power across slices and subjects. Within a range from 0.015 Hz to 0.075 Hz, ventral GM exhibits 32% more power than WM whereas dorsal GM exhibits only 5% more power. This difference in power between GM sub-regions may be explained through the preprocessing pipeline permutations presented in Figure 5—figure supplement 1. Figure 5—figure supplement 1A,B show comparable *z*-scores between ventral and dorsal GM horns, and this difference does not appear until after WM regression (Figure 5—figure supplement 1C) or WM&GM regression (Figure 5—figure supplement 1D). Therefore, regression with this final eigenvector suppresses a larger contribution of signal fluctuations (and thus power) from dorsal GM than ventral GM, which may be attributed to the small size of the dorsal horns and partial volume averaging effects (because the WM mask is not eroded before PCA), as well as to unavoidable sub-millimeter registration inaccuracies. The large peak at ∼0.75 Hz is likely due to physiological noise, so it could be argued that this noise power significantly affects the measurements of functional connectivity. To investigate this further and discount the possibility that our results are driven by physiological noise around 0.75 Hz, the analyses in Figure 5—figure supplement 1 are repeated after data are filtered with a narrower band-pass filter between 0.01 Hz and 0.07 Hz. These results are presented in Figure 5—figure supplement 3. **DOI:** http://dx.doi.org/10.7554/eLife.02812.010

**Figure 5—figure supplement 3.**
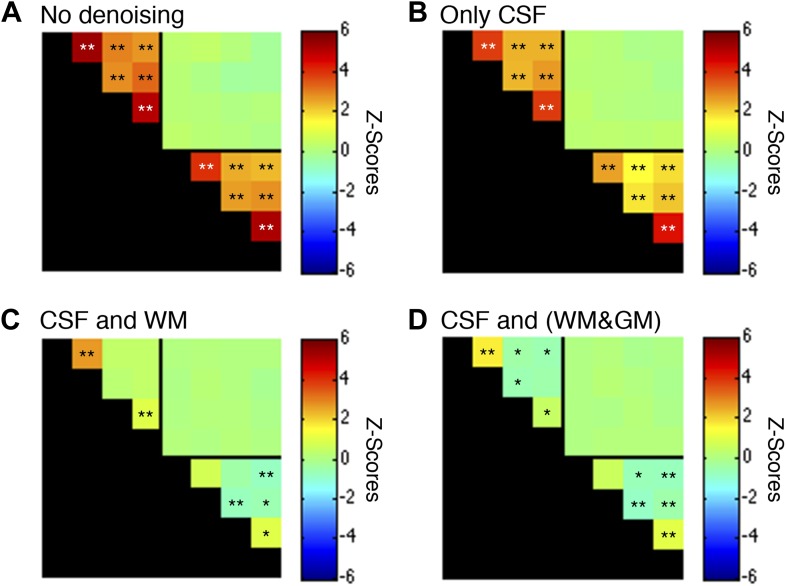
Functional connectivity matrices resulting from preprocessing pipeline permutations after band-pass filtering between 0.01 and 0.07 Hz. Functional connectivity matrices resulting from preprocessing pipeline permutations after band-pass filtering between 0.01 and 0.07 Hz to suppress power from likely physiological noise at ∼0.75 Hz (*p<0.05; **p<0.01; Bonferroni corrected). For clarity the labels are not shown for each column/row but are the same as in Figure 5. (**A**) Preprocessing was performed as described in the Methods except CSF and WM regressors (steps #11 and #12) were not applied, and step #13 used a different frequency bandwidth. (**B**) Preprocessing was performed as described in ‘Materials and methods’ except a WM regressor (step #12) was not applied and step #13 used a different frequency bandwidth. (**C**) Preprocessing was performed as described in ‘Materials and methods’ except step #13 used a different frequency bandwidth. (**D**) Preprocessing was performed as described in ‘Materials and methods’ except step #12 extracted the principal eigenvector of all time series within a combined WM and GM mask and step #13 used a different frequency bandwidth. Only a couple of minor changes are apparent between these matrices and those presented in Figure 5—figure supplement 1, suggesting that a slight decrease in the upper filter range from 0.08 Hz to 0.07 Hz to further suppress physiological noise does not appear to have a significant impact on the group-level connectivity analyses. Power spectra for WM and GM sub-regions in (**C**) are presented in Figure 5—figure supplement 4. **DOI:** http://dx.doi.org/10.7554/eLife.02812.011

**Figure 5—figure supplement 4.**
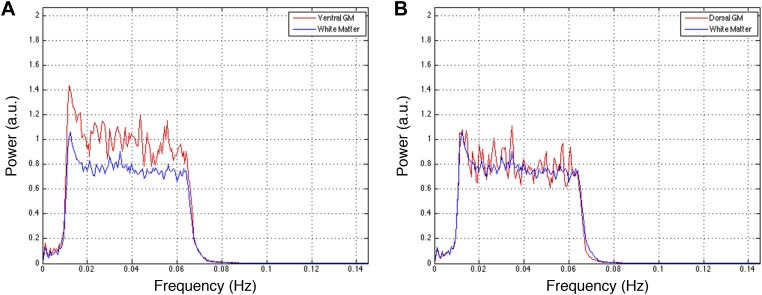
Power spectra across gray and white matter sub-regions for data filtered between 0.01 and 0.07 Hz. Power spectra for (**A**) ventral and (**B**) dorsal GM sub-regions in Figure 5—figure supplement 3C that exhibit significant positive correlations in the original analysis (Figure 5A; *z* > 1.65; one-tailed), and all WM sub-regions. For each frequency the plotted power represents median power across slices and subjects. Within a range from 0.015 Hz to 0.065 Hz, ventral GM similarly exhibits 31% more power than WM whereas dorsal GM exhibits 5% more power. The narrower bandwidth completely suppresses the noise peak at ∼0.75 Hz but the results from functional connectivity analyses are almost unchanged (Figure 5—figure supplement 1 vs Figure 5—figure supplement 3), confirming that this peak does not significantly affect the overall results. Our original decision to filter resting state spinal cord data between 0.01 Hz and 0.08 Hz (step #13) was motivated by the approach most commonly used for resting state analyses in the brain (filtering between 0.01 Hz and 0.08–0.1 Hz), although it is not yet clear if this frequency range is optimal for resting state spinal cord analyses. To investigate the possibility that frequencies above 0.08 Hz may contribute to inherent functional connectivity, the analyses in Figure 5—figure supplement 1 are once again repeated after data are filtered with a wider band-pass filter between 0.01 Hz and 0.13 Hz. These results are presented in Figure 5—figure supplement 5. **DOI:** http://dx.doi.org/10.7554/eLife.02812.012

**Figure 5—figure supplement 5.**
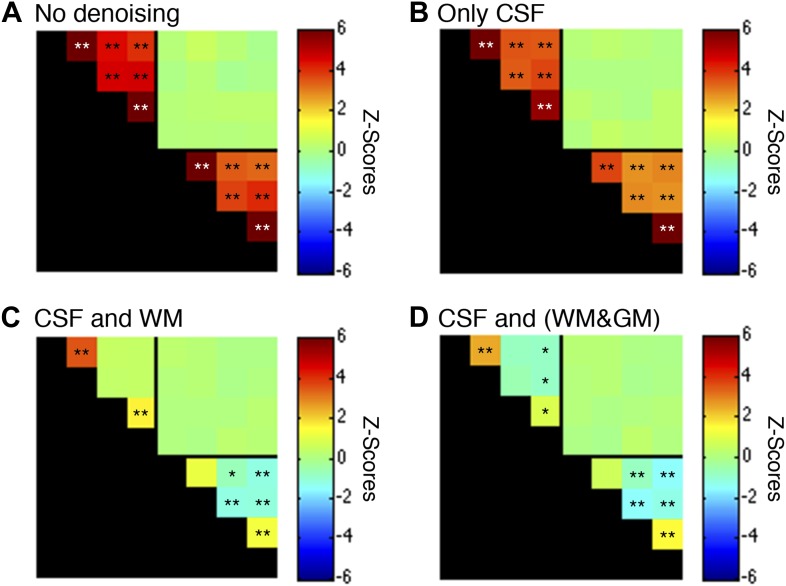
Functional connectivity matrices resulting from preprocessing pipeline permutations after band-pass filtering between 0.01 and 0.13 Hz. Functional connectivity matrices resulting from preprocessing pipeline permutations after band-pass filtering between 0.01 and 0.13 Hz (*p<0.05; **p<0.01; Bonferroni corrected). For clarity the labels are not shown for each column/row but are the same as in Figure 5. (**A**) Preprocessing was performed as described in the Methods except CSF and WM regressors (steps #11 and #12) were not applied, and step #13 used a different frequency bandwidth. (**B**) Preprocessing was performed as described in ‘Materials and methods’ except a WM regressor (step #12) was not applied and step #13 used a different frequency bandwidth. (**C**) Preprocessing was performed as described in ‘Materials and methods’ except step #13 used a different frequency bandwidth. (**D**) Preprocessing was performed as described in ‘Materials and methods’ except step #12 extracted the principal eigenvector of all time series within a combined WM and GM mask and step #13 used a different frequency bandwidth. The inclusion of frequencies between 0.08 Hz and 0.13 Hz primarily strengthens GM correlations between ventral horns (relative to the results obtained using only frequencies between 0.01 Hz and 0.08 Hz) but also increases the statistical significance of WM correlations between LD and RD. Power spectra for WM and GM sub-regions in (**C**) are presented in Figure 5—figure supplement 6. **DOI:** http://dx.doi.org/10.7554/eLife.02812.013

**Figure 5—figure supplement 6.**
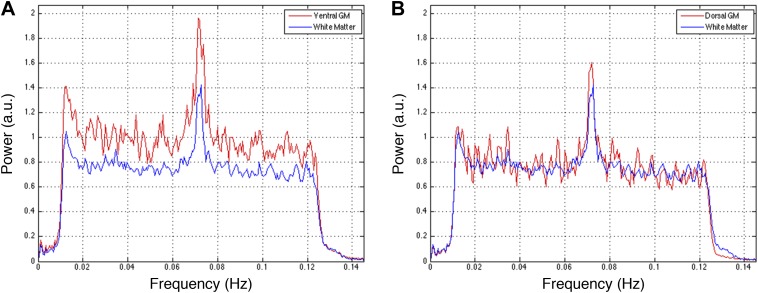
Power spectra across gray and white matter sub-regions for data filtered between 0.01 and 0.13 Hz. Power spectra for (**A**) ventral and (**B**) dorsal GM sub-regions in Figure 5—figure supplement 5C that exhibit significant positive correlations in the original analysis (Figure 5A; *z* > 1.65; one-tailed), and all WM sub-regions. For each frequency the plotted power represents median power across slices and subjects. Within the range of higher frequencies between 0.075 Hz and 0.125 Hz, ventral GM exhibits 28% more power than WM whereas dorsal GM and WM exhibit comparable total power (< 0.3% difference). These data show that the original filter bandwidth between 0.01 Hz and 0.08 Hz was a sound choice, but also suggest that frequencies slightly above 0.08 Hz may contain additional power related to GM connectivity. Future work will acquire resting state spinal cord data with a faster sampling rate to better understand the relative frequency-dependent contributions from BOLD signal fluctuations and physiological noise. **DOI:** http://dx.doi.org/10.7554/eLife.02812.014

## Discussion

We have presented the first conclusive demonstration that ultra-high field fMRI can non-invasively detect and characterize resting state BOLD signals within the gray matter of the human spinal cord. Low-frequency temporal correlations were observed between gray matter horns in all subjects, and examples of these correlations are presented in Figure 3 and Figure 4. Within a given axial slice, the strongest and most robust correlations were between left and right ventral horns. Correlations were also observed between left and right dorsal horns, and the reproducibility of correlations between ventral horns and between dorsal horns was demonstrated within a cohort of 22 healthy volunteers (Figure 5). Although correlations between ventral and dorsal gray matter were also observed at the single-subject level (Figure 4L), such findings were less frequent and not statistically significant at the group level. The absence of group-level correlations between spinal gray matter and adjacent white matter showed that these positive gray matter correlations are unlikely to be driven by spatially-correlated physiological noise. In fact, supplementary analyses without CSF or white matter regressors (Figure 5—figure supplement 1a) revealed strong correlations within gray matter and within white matter but not between gray and white matter, suggesting that gray and white matter in the spinal cord may exhibit different degrees of physiological fluctuations. The main analyses (Figure 5) also showed anticorrelations between white matter sub-regions. These observations were not unexpected because negative correlations are commonly seen in the brain (Chang and Glover, 2010), but additional analyses (Figure 5—figure supplement 1) revealed that correlations within white matter were heavily influenced by preprocessing methodology. As a result, the nature of negative white matter correlations remains unclear and requires further investigation. However, predominantly positive correlations between dorsal white matter sub-regions persisted across preprocessing permutations. Although the origins of these positive white matter correlations remain to be determined, and whereas in general BOLD signals from activation have been difficult to detect in brain white matter, we note that Ding et al. recently reported the reliable detection of anisotropic correlations of resting state BOLD signals in brain white matter that appear to mimic white matter tracts identified by diffusion imaging methods (Ding et al., 2013). Moreover, the white matter sub-regions within the cord also tend to be close to draining veins.

Another observation is that *z*-scores measured between ventral horns tended to be higher than *z*-scores measured between dorsal horns—a finding that was highly significant (p < 0.01 using a two-tailed Wilcoxon signed rank test; ‘signrank’ in Matlab). There are several possible reasons for this finding. Firstly, the dorsal horns tend to be slightly narrower than the ventral horns, and thus may be more susceptible to registration inaccuracies, residual physiological noise, and partial volume averaging with adjacent white matter. Secondly, as shown in Figure 2, signal dropout and unavoidable artifacts affect the dorsal horns but not ventral horns in a few slices, which would bias the results in favor of ventral horn connectivity. Finally, even if spatial artifacts, registration inaccuracies, signal dropout, physiological noise, and partial volume averaging effects were minimized, it may be that functional connectivity between dorsal horns is more variable if the automated selection of gray matter sub-regions (step #14) isolate signals from different laminae within dorsal horns (Ruscheweyh and Sandkühler, 2002).

To date, clinical applications of task-based spinal fMRI studies have primarily been targeted to subjects with multiple sclerosis (Agosta et al., 2008a, 2008b, 2009b; Valsasina et al., 2010, 2012) and spinal cord injury (SCI) (Stroman et al., 2002, 2004; Kornelsen and Stroman, 2007). We propose that the non-invasive methods of resting state spinal cord functional connectivity developed in this paper may be most readily translatable to clinical investigations characterizing damage due to acute or chronic SCI and monitoring the efficacy of surgical or pharmacological interventions. Functional connectivity and the assessment of plasticity in the human spinal cord and animal models of SCI have been topics of intense research for many years (Bregman et al., 1997; Raineteau and Schwab, 2001; Cai et al., 2006; Freund et al., 2011) because SCI affects 260,000 people in the United States (a prevalence of ∼1 in 1200) with 11,000 new injuries reported each year (National Spinal Cord Injury Statistics Center, 2010). Studies have investigated the role of propriospinal neurons in partial recovery from incomplete SCI (Bareyre, 2008; Flynn et al., 2011a) while in vitro analyses of functional connectivity in mouse spinal cord have relied on electrophysiological methods (Flynn et al., 2011b). A new intervention strategy using epidural stimulation and stand training was recently developed that has to date restored voluntary movement in four patients with complete paralysis, demonstrating that functional connectivity across a lesion may be restored with epidural stimulation (Angeli et al., 2014). Such studies would likely benefit from an ability to assess the functional architecture of the spinal cord throughout therapy. The majority of spinal injuries are, however, incomplete, and lost function may eventually return to near-normal levels; however, the progression of functional recovery after incomplete SCI remains poorly understood due to the absence of a non-invasive method to reliably assess spinal cord connectivity in vivo. We propose that resting state acquisitions of the cervical spinal cord will become a valuable tool for characterizing changes in functional connectivity in SCI, and for the prognosis and monitoring of progression of recovery via spontaneous repair and/or surgical intervention. For example, the imaging volume (shown in Figure 1A) may be centered on a focal injury to the cord, and functional connectivity above and below the injury may be assessed. This process with identical slice placement could then be repeated serially over time to investigate phenomena of neural plasticity and adaptation of spinal pathways. Moreover, even in normal subjects, the functional organization of the spinal cord is relatively under-explored and remains poorly understood. Observations of altered resting state connectivity in the brain in numerous disorders (Fox and Greicius, 2010) and as a function of behavior or cognitive skills suggest that such correlations reflect an important level of organization and may play a fundamental role in the execution and maintenance of various functions (Pizoli et al., 2011). Thus, investigation of resting state spinal cord networks could similarly have widespread applicability in studying central nervous system diseases that affect motor and/or sensory pathways such as cervical spondylotic myelopathy, neuromyelitis optica, acute disseminated encephalomyelitis, arachnoiditis, transverse myelitis, amyotrophic lateral sclerosis, and multiple sclerosis.

Our study has three main limitations. Firstly, although we observed statistically significant correlations along the cord in single-subject analyses (for example, in left ventral gray matter in Figure 3), we chose to constrain the group analysis to investigating connectivity within each axial slice. This was done because incorporating correlations along the cord would have made the analysis significantly more complicated by increasing the number of potential correlations by an order of magnitude. Furthermore, as shown in Figure 2, some slices exhibited regions of signal dropout at the dorsal edge caused by fat shift of the nerve root sleeve in the phase-encode (anterior-posterior) direction. The absence of a statistically significant correlation with a slice impacted by main field (B_0_) inhomogeneities or an artifact cannot in and of itself be interpreted as proof that connectivity does not exist between particular sub-regions of interest, and ultimately very careful single-subject analyses will need to be performed to reliably characterize functional connectivity along the cord. Secondly, although we have investigated one aspect of reproducibility (via a group analysis of 22 healthy volunteers) and established the existence of spinal cord functional connectivity, further investigations of within-subject reproducibility still need to be performed. This could be done by acquiring multiple resting state runs from a single scanning session and/or by scanning the same volunteer on multiple days while ensuring that the imaging volume is consistently placed in the same location (e.g., centered on the C3/C4 junction, as shown in Figure 1A). The reproducibility of these connectivity measures over months and years will need to be quantified before such techniques can be reliably used to study disease progression or gerontology. Thirdly, this study reports empirical findings of functional connectivity in the spinal cord but does not directly address the physiological origins of these low-frequency BOLD signal fluctuations. However, as a point of reference, observations of functional connectivity were first reported in the brain nearly two decades ago (Biswal et al., 1995) and yet the origin of these fluctuations is still a topic of intense discussion. Although the nature of resting state correlations is not fully understood, recent studies have combined tractography and functional imaging with techniques such as network analysis and graph theory to explore how these networks may have emerged (Deco et al., 2013a, 2013b; van den Heuvel and Sporns, 2013; Mišić et al., 2014; Goñi et al., 2014). The origin of resting state networks has also been explored within the context of evolution and the expansion of the cerebral cortex (Buckner and Krienen, 2013). We therefore propose that because the spinal cord is an integral part of the central nervous system, one simple explanation is that low-frequency BOLD fluctuations in the brain and spinal cord share the same origin. This theory would then suggest that there may be long-range connections between networks in the spinal cord and cerebrum (and cerebellum), and future research should consider the nature of resting state networks not only within the brain but within the entire central nervous system.

Previous attempts to detect functional connectivity in the spinal cord have undoubtedly been confronted by significant technical challenges. At conventional field strengths (1.5 Tesla and 3.0 Tesla), the lower signal-to-noise ratio and BOLD contrast limit the spatial resolution and sensitivity for detecting BOLD signals, so large voxels are typically used and multiple acquisitions are averaged. At 7 Tesla, high resolution BOLD acquisitions of the spinal cord have not evolved as rapidly as for the brain in part due to the widespread dependence on single-shot echo-planar acquisitions at lower fields and the lack of specialized coils to image the spinal cord. We addressed these challenges and limitations by using a 7 Tesla scanner, novel fMRI image acquisition and data correction protocols, and a dedicated 16-channel radiofrequency coil array designed for cord imaging. Our results are the first demonstrations of functional imaging of the human spinal cord at 7 Tesla (Barry et al., 2013a) and high-resolution resting state functional connectivity in the spinal cord (Barry et al., 2013b), and are likely to be of significant relevance in understanding basic aspects of spinal cord function both in normal development and in clinical disorders of the central nervous system.

## Materials and methods

### Data acquisition

Experiments were performed on a Philips Achieva 7 Tesla scanner with a custom-designed (Nova Medical Inc.) quadrature transmit and 16-channel receive coil array for cervical spinal cord imaging. 22 healthy volunteers (11 male, 21–63 years; 11 female, 23–34 years; 28.4 ± 8.8 years) with no history of spinal cord injury or neurological impairment were recruited and scanned under protocols approved by the Institutional Review Board at Vanderbilt University Medical Center. Female participants of childbearing potential required a negative urine pregnancy test for the scan to proceed. Non-MR study data were collected and managed using REDCap electronic data capture tools hosted at Vanderbilt University (Harris et al., 2009). REDCap (Research Electronic Data Capture) is a secure, web-based application designed to support data capture for research studies.

Anatomical axial images with high spatial resolution and T_2_^*^-weighting (Figure 1B) were acquired with the following MR parameters: field of view = 160 × 160 mm^2^, 12 4-mm slices (centered on the C3/C4 junction, as shown in Figure 1A), nominal voxel size = 0.6 × 0.6 × 4 mm^3^, interpolated voxel size = 0.31 × 0.31 × 4 mm^3^, repetition time = 303 ms, echo time = 8.2 ms, flip angle = 25°, sensitivity encoding (SENSE) (Pruessmann et al., 1999) reduction factor = 2.0 (anterior-posterior), signal acquisitions = 8, total acquisition time = 5 min 22 s.

Functional images with identical slice placement were acquired with a 3D multi-shot gradient-echo sequence (van der Meulen et al., 1988) previously shown to minimize T_2_^*^ blurring and geometric distortions in cortical fMRI at 7 Tesla (Barry et al., 2011). The functional MR parameters for the first 11 subjects were: field of view = 160 × 160 mm, twelve 4-mm slices, voxel size = 0.91 × 0.91 × 4 mm^3^, repetition time = 18 ms, echo time = 7.8 ms, flip angle = 15°, echo train length = 9, SENSE reduction factor = 1.56 (anterior-posterior), volume acquisition time = 3.6 s (300 ms/slice), number of volumes = 150 (after 10 ‘dummy’ scans), total scan time = 9 min 38 s, max gradient strength = 30 mT/m, max slew rate = 175 mT/m/ms. The functional MR parameters for the last 11 subjects were the same except for the following minor adjustments: repetition time = 17 ms, echo time = 8.0 ms, volume acquisition time = 3.34 s (278 ms/slice), total scan time = 9 min. For all subjects, respiratory and cardiac cycles were externally monitored and recorded using a respiratory bellow (placed on the abdomen) and pulse oximeter (placed on the left index finger).

### Data processing

Functional data were corrected for physiological noise using methods that are commonly used in fMRI of the brain in addition to novel data-driven ‘regressors of no interest’. The steps applied to all spinal fMRI data are as follows:

1. For each slice of anatomical and functional images, a 2D Gaussian weighting kernel was manually defined with the full-width-at-half-maximum set at the CSF boundaries. Weighting masks defined on anatomical images were used in affine registration (step #8), and weighting masks defined on functional images were used in affine registration as well as rigid-body motion correction (step #5).
2. For each slice, a ‘not-spine’ mask was defined by drawing a region around the entire spinal cord and then logically inverting it (used in step #3).
3. For each slice, data-driven ‘regressors of no interest’ were selected via principal component analysis (PCA) of all voxels within the not-spine mask to identify structured noise sources that would similarly affect the spinal cord and external (neck) regions. The number of eigenvectors selected reflected up to 80% of the slice-wise cumulative variance or until the difference between two successive eigenvalues was less than 2% (typically 3–5 per slice). These vectors were regressed from the time series of all voxels within a slice, and significantly improved the efficacy of motion correction (step #5) by mitigating widespread intensity fluctuations due to physiological processes (e.g., swallowing).
4. For each slice, a representative (target) volume was automatically selected for motion correction by calculating the median intensity of each voxel (over time) and then selecting the volume closest to the median image (identified via minimal least squares error).
5. Rigid-body motion correction was performed on a slice-wise basis (using 3dWarpDrive in AFNI [Cox, 1996]) using the target volumes (identified in step #4). Motion was constrained to be within-plane translation (i.e., no rotation of the spinal cord). To mitigate the detrimental effects of sporadic artifacts (e.g., swallowing) on motion parameter estimation, translation estimates were filtered with a 5-point median filter and then re-applied (using 3dAllineate in AFNI [Cox, 1996]) to the original data before motion correction. The initial registration (to obtain motion parameter estimates) used quintic interpolation and the final transformation used sinc interpolation.
6. An established image correction technique called RETROICOR [Glover et al., 2000] (implemented in AFNI [Cox, 1996]) was applied to the entire functional volume to further reduce quasi-periodic intensity variations due to physiological noise.
7. Using the high-resolution anatomical images as a reference, masks defining the boundaries of gray matter, white matter, and CSF were created for each slice.
8. Affine registration of the target volume (identified in step #4) to the anatomical image was performed (via 3dAllineate) on a slice-wise basis. The Hellinger metric was selected as the cost function, and the degrees of freedom were constrained to within-plane translation, scaling (maximum of 1% in the read direction and 5% in the phase-encode direction), and shearing (maximum of 5%).
9. The affine transforms (defined in step #8) were applied to all functional volumes (via 3dAllineate), and transformed functional images were resampled (with sinc interpolation) to match the final resolution of the anatomical volume (voxel size = 0.31 × 0.31 × 4 mm^3^).
10. The quality of the final functional-to-anatomical alignments was visually verified using MRIcron (www.mccauslandcenter.sc.edu/mricro/mricron).
11. For each slice, additional data-driven ‘regressors of no interest’ were selected via PCA of all functional voxels within the CSF mask (defined in step #7) to identify structured noise sources that would similarly affect gray matter and CSF. The number of eigenvectors selected reflected up to 50% of the slice-wise cumulative variance or until the difference between two successive eigenvalues was less than 2% (typically 2–6 per slice). These vectors were regressed from the time series of all spinal cord voxels within a slice.
12. For each slice, a ‘global’ white matter signal was calculated via PCA of all functional voxels within the white matter mask and extraction of the first eigenvector (typically representing 10–30% of the variance). This was primarily done to mitigate any residual variance due to shifting of the white matter boundary (caused by motion) but would also reduce variance caused by residual physiological noise. This vector was regressed from the time series of all gray and white matter voxels within a slice.
13. In preparation for analyses of functional connectivity, resultant functional data were band-pass filtered between 0.01 and 0.08 Hz using a Chebyshev Type II filter (‘cheby2’ and ‘filtfilt’ in Matlab) to emphasize low-frequency signals of interest.
14. In preparation for group analyses, gray and white matter masks (defined in step #7) were subdivided into quadrants to identify left and right ventral and dorsal horns (excluding central gray matter connecting left and right sides), as well as the four adjacent white matter regions. Each of these eight sub-region masks (per slice) was morphologically eroded (using ‘imerode’ in Matlab) to remove the outermost voxels and mitigate partial volume effects. For gray matter the morphological eroding object was a disk with a radius of 3 voxels, and for white matter it was a disk with a radius of 11 voxels. If the eroded sub-region did not contain any voxels (i.e., the disk was too large) then the disk size was incrementally decreased by 1 voxel, and the erosion process repeated until the innermost area of each sub-region was extracted. Time series extracted from each eroded sub-region were also used in single-subject analyses (except for in Figure 3 and Figure 4, which used single-voxel correlations) and power spectra calculations (figure supplements for Figure 5).

Figure supplements for Figure 5 investigate modifications to steps #11, #12, and/or #13, and deviations from this standardized preprocessing pipeline are described in the respective figure legends.

### Data analysis

For the voxel-based analysis shown in Figure 3, the linear correlation coefficient (*r*) was calculated between a seed voxel and all other voxels in Matlab. These correlation values were converted to *z*-scores using the Fisher *r*-to-*z* transformation *z* = tanh^−1^(*r*)(*dof*–3)^1/2^ where *dof* is the estimated degrees of freedom for each voxel after correction for first-order autocorrelation (Rogers and Gore, 2008). A statistical threshold of |*z*| > 3.29 (a two-tailed 99.9% confidence interval) was selected to clearly show that gray matter correlations tend to be focused in the center of the horns. A minimum cluster threshold of nine contiguous interpolated voxels (3.49 mm^3^, approximately equal to the native functional voxel volume of 3.31 mm^3^) was also used to further protect against spurious correlations.

For the single-subject analyses presented in Figure 4, AFNI's ‘InstaCorr’ (Cox, 1996) was used to display correlations between a single gray matter voxel and all other voxels within the spinal cord. The correlation threshold was p < 0.001 and in these analyses no cluster thresholding was used to reveal all correlations within gray and white matter.

Finally, for the group-level region-of-interest (ROI)-based analyses (Figure 5), the time series of individual voxels were averaged within each eroded mask (created in step #14) for each subject. The linear correlation coefficient was then calculated between the averaged time course from a sub-region and all other sub-regions. These correlation coefficients were converted to *z*-scores and corrected for first-order autocorrelation (Rogers and Gore, 2008). For each ROI comparison (e.g., left dorsal horn vs right dorsal horn), the median *z*-score (unthresholded) was calculated across all 12 slices for each of the 22 subjects, and then a two-tailed Wilcoxon signed rank test (‘signrank’ in Matlab) identified group-level distributions of 22 *z*-scores that were significantly different from zero (p < 0.05 or p < 0.01 with a Bonferroni correction factor of 28).

## References

[bib1] Agosta F Valsasina P Caputo D Stroman PW Filippi M 2008a Tactile-associated recruitment of the cervical cord is altered in patients with multiple sclerosis. Neuroimage 39:1542–1548. doi: 10.1016/j.neuroimage.2007.10.048 18061484

[bib2] Agosta F Valsasina P Rocca MA Caputo D Sala S Judica E Stroman PW Filippi M 2008b Evidence for enhanced functional activity of cervical cord in relapsing multiple sclerosis. Magnetic Resonance in Medicine 59:1035–1042. doi: 10.1002/mrm.21595 18429010

[bib3] Agosta F Valsasina P Caputo D Rocca MA Filippi M 2009a Tactile-associated fMRI recruitment of the cervical cord in healthy subjects. Human Brain Mapping 30:340–345. doi: 10.1002/hbm.20499 18041739 PMC6870728

[bib4] Agosta F Valsasina P Absinta M Sala S Caputo D Filippi M 2009b Primary progressive multiple sclerosis: tactile-associated functional MR activity in the cervical spinal cord. Radiology 253:209–215. doi: 10.1148/radiol.2532090187 19703852

[bib5] Angeli CA Edgerton VR Gerasimenko YP Harkema SJ 2014 Altering spinal cord excitability enables voluntary movements after chronic complete paralysis in humans. Brain 137:1394–1409. doi: 10.1093/brain/awu038 24713270 PMC3999714

[bib7] Backes WH Mess WH Wilmink JT 2001 Functional MR imaging of the cervical spinal cord by use of median nerve stimulation and fist clenching. AJNR. American Journal of Neuroradiology 22:1854–1859 11733315 PMC7973824

[bib8] Bandettini PA Wong EC Hinks RS Tikofsky RS Hyde JS 1992 Time course EPI of human brain function during task activation. Magnetic Resonance in Medicine 25:390–397. doi: 10.1002/mrm.1910250220 1614324

[bib9] Bareyre FM 2008 Neuronal repair and replacement in spinal cord injury. Journal of the Neurological Sciences 265:63–72. doi: 10.1016/j.jns.2007.05.004 17568612

[bib10] Barry RL Strother SC Gatenby JC Gore JC 2011 Data-driven optimization and evaluation of 2D EPI and 3D PRESTO for BOLD fMRI at 7 Tesla: I. Focal coverage. Neuroimage 55:1034–1043. doi: 10.1016/j.neuroimage.2010.12.086 21232613 PMC3049844

[bib11] Barry RL Gore JC Smith SA 2013a Development of BOLD-sensitive protocols for imaging the human spinal cord at 7 Tesla. Proceedings of the International Society for Magnetic Resonance in Medicine 21:2278

[bib12] Barry RL Smith SA Gore JC 2013b Resting state functional connectivity in the human spinal cord at 7 Tesla. Proceedings of the International Society for Magnetic Resonance in Medicine 21:2237

[bib13] Biswal B Yetkin FZ Haughton VM Hyde JS 1995 Functional connectivity in the motor cortex of resting human brain using echo-planar MRI. Magnetic Resonance in Medicine 34:537–541. doi: 10.1002/mrm.1910340409 8524021

[bib14] Bregman BS Diener PS McAtee M Dai HN James C 1997 Intervention strategies to enhance anatomical plasticity and recovery of function after spinal cord injury. Advances in Neurology 72:257–275 8993704

[bib15] Brooks JCW Kong Y Lee MC Warnaby CE Wanigasekera V Jenkinson M Tracey I 2012 Stimulus site and modality dependence of functional activity within the human spinal cord. The Journal of Neuroscience 32:6231–6239. doi: 10.1523/JNEUROSCI.2543-11.2012 22553029 PMC6622146

[bib16] Buckner RL Krienen FM 2013 The evolution of distributed association networks in the human brain. Trends in Cognitive Sciences 17:648–665. doi: 10.1016/j.tics.2013.09.017 24210963

[bib17] Cai LL Courtine G Fong AJ Burdick JW Roy RR Edgerton VR 2006 Plasticity of functional connectivity in the adult spinal cord. Philosophical Transactions of the Royal Society of London. Series B, Biological Sciences 361:1635–1646. doi: 10.1098/rstb.2006.1884 PMC1664672 16939979

[bib18] Chang C Glover GH 2010 Time-frequency dynamics of resting-state brain connectivity measured with fMRI. Neuroimage 50:81–98. doi: 10.1016/j.neuroimage.2009.12.011 20006716 PMC2827259

[bib19] Cohen-Adad J Gauthier CJ Brooks JCW Slessarev M Han J Fisher JA Rossignol S Hoge RD 2010 BOLD signal responses to controlled hypercapnia in human spinal cord. Neuroimage 50:1074–1084. doi: 10.1016/j.neuroimage.2009.12.122 20060914

[bib20] Cox RW 1996 AFNI: software for analysis and visualization of functional magnetic resonance neuroimages. Computers and Biomedical Research 29:162–173. doi: 10.1006/cbmr.1996.0014 8812068

[bib21] Deco G Jirsa VK McIntosh AR 2013a Resting brains never rest: computational insights into potential cognitive architectures. Trends in Neurosciences 36:268–274. doi: 10.1016/j.tins.2013.03.001 23561718

[bib22] Deco G Ponce-Alvarez A Mantini D Romani GL Hagmann P Corbetta M 2013b Resting-state functional connectivity emerges from structurally and dynamically shaped slow linear fluctuations. The Journal of Neuroscience 33:11239–11252. doi: 10.1523/JNEUROSCI.1091-13.2013 23825427 PMC3718368

[bib23] Ding Z Newton AT Xu R Anderson AW Morgan VL Gore JC 2013 Spatio-temporal correlation tensors reveal functional structure in human brain. PLOS ONE 8:e82107. doi: 10.1371/journal.pone.0082107 24339997 PMC3855380

[bib24] Flynn JR Graham BA Galea MP Callister RJ 2011a The role of propriospinal interneurons in recovery from spinal cord injury. Neuropharmacology 60:809–822. doi: 10.1016/j.neuropharm.2011.01.016 21251920

[bib25] Flynn JR Brichta AM Galea MP Callister RJ Graham BA 2011b A horizontal slice preparation for examining the functional connectivity of dorsal column fibres in mouse spinal cord. Journal of Neuroscience Methods 200:113–120. doi: 10.1016/j.jneumeth.2011.06.017 21726580

[bib26] Fox MD Greicius M 2010 Clinical applications of resting state functional connectivity. Frontiers in Systems Neuroscience 4:1–13. doi: 10.3389/fnsys.2010.00019 20592951 PMC2893721

[bib27] Fox MD Snyder AZ Vincent JL Corbetta M Van Essen DC Raichle ME 2005 The human brain is intrinsically organized into dynamic, anticorrelated functional networks. Proceedings of the National Academy of Sciences of the United States of America 102:9673–9678. doi: 10.1073/pnas.0504136102 15976020 PMC1157105

[bib28] Freund P Weiskopf N Ward NS Hutton C Gall A Ciccarelli O Craggs M Friston K Thompson AJ 2011 Disability, atrophy and cortical reorganization following spinal cord injury. Brain: a Journal of Neurology 134:1610–1622. doi: 10.1093/brain/awr093 21586596 PMC3102242

[bib29] Giove F Garreffa G Giulietti G Mangia S Colonnese C Maraviglia B 2004 Issues about the fMRI of the human spinal cord. Magnetic Resonance Imaging 22:1505–1516. doi: 10.1016/j.mri.2004.10.015 15707800

[bib30] Giulietti G Giove F Garreffa G Colonnese C Mangia S Maraviglia B 2008 Characterization of the functional response in the human spinal cord: impulse-response function and linearity. Neuroimage 42:626–634. doi: 10.1016/j.neuroimage.2008.05.006 18599318

[bib31] Glover GH Li TQ Ress D 2000 Image-based method for retrospective correction of physiological motion effects in fMRI: RETROICOR. Magnetic Resonance in Medicine 44:162–167. doi: 10.1002/1522-2594(200007)44:13.0.CO;2-E 10893535

[bib32] Goñi J van den Heuvel MP Avena-Koenigsberger A Velez de Mendizabal N Betzel RF Griffa A Sporns O 2014 Resting-brain functional connectivity predicted by analytic measures of network communication. Proceedings of the National Academy of Sciences of the United States of America 111:833–838. doi: 10.1073/pnas.1315529111 24379387 PMC3896172

[bib33] Greicius MD Krasnow B Reiss AL Menon V 2003 Functional connectivity in the resting brain: a network analysis of the default mode hypothesis. Proceedings of the National Academy of Sciences of the United States of America 100:253–258. doi: 10.1073/pnas.0135058100 12506194 PMC140943

[bib34] Harris PA Taylor R Thielke R Payne J Gonzalez N Conde JG 2009 Research electronic data capture (REDCap)―A metadata-driven methodology and workflow process for providing translational research informatics support. Journal of Biomedical Informatics 42:377–381. doi: 10.1016/j.jbi.2008.08.010 18929686 PMC2700030

[bib35] Kandel ER Schwartz JH Jessell TM 2000 Principles of Neural Science. 4th edition McGraw-Hill

[bib36] Kornelsen J Stroman PW 2007 Detection of the neuronal activity occurring caudal to the site of spinal cord injury that is elicited during lower limb movement tasks. Spinal Cord 45:485–490. doi: 10.1038/sj.sc.3102019 17245349

[bib37] Kwong KK Belliveau JW Chesler DA Goldberg IE Weisskoff RM Poncelet BP Kennedy DN Hoppel BE Cohen MS Turner R Cheng HM Brady TJ Rosen BR 1992 Dynamic magnetic resonance imaging of human brain activity during primary sensory stimulation. Proceedings of the National Academy of Sciences of the United States of America 89:5675–5679. doi: 10.1073/pnas.89.12.5675 1608978 PMC49355

[bib38] Madi S Flanders AE Vinitski S Herbison GJ Nissanov J 2001 Functional MR imaging of the human cervical spinal cord. AJNR. American Journal of Neuroradiology 22:1768–1774 11673177 PMC7974439

[bib39] Maieron M Iannetti GD Bodurka J Tracey I Bandettini PA Porro CA 2007 Functional responses in the human spinal cord during willed motor actions: evidence for side- and rate-dependent activity. The Journal of Neuroscience 27:4182–4190. doi: 10.1523/JNEUROSCI.3910-06.2007 17428996 PMC6672553

[bib40] Mišić B Sporns O McIntosh AR 2014 Communication efficiency and congestion of signal traffic in large-scale brain networks. PLOS Computational Biology 10:e1003427. doi: 10.1371/journal.pcbi.1003427 24415931 PMC3886893

[bib41] Moffitt MA Dale BM Duerk JL Grill WM 2005 Functional magnetic resonance imaging of the human lumbar spinal cord. Journal of Magnetic Resonance Imaging: JMRI 21:527–535. doi: 10.1002/jmri.20314 15834921

[bib6] National Spinal Cord Injury Statistics Center (NSCISC) 2010 Annual report for the spinal cord injury model systems public version.

[bib42] Ogawa S Lee TM Kay AR Tank DW 1990 Brain magnetic resonance imaging with contrast dependent on blood oxygenation. Proceedings of the National Academy of Sciences of the United States of America 87:9868–9872. doi: 10.1073/pnas.87.24.9868 2124706 PMC55275

[bib43] Ogawa S Tank DW Menon R Ellermann JM Kim S-G Merkle H Ugurbil K 1992 Intrinsic signal changes accompanying sensory stimulation: functional brain mapping with magnetic resonance imaging. Proceedings of the National Academy of Sciences of the United States of America 89:5951–5955. doi: 10.1073/pnas.89.13.5951 1631079 PMC402116

[bib44] Pizoli CE Shah MN Snyder AZ Shimony JS Limbrick DD Raichle ME Smyth MD 2011 Resting-state activity in development and maintenance of normal brain function. Proceedings of the National Academy of Sciences of the United States of America 108:11638–11643. doi: 10.1073/pnas.1109144108 21709227 PMC3136307

[bib45] Pruessmann KP Weiger M Scheidegger MB Boesiger P 1999 SENSE: sensitivity encoding for fast MRI. Magnetic Resonance in Medicine: Official Journal of the Society of Magnetic Resonance in Medicine/Society of Magnetic Resonance in Medicine 42:952–962. doi: 10.1002/(SICI)1522-2594(199911)42:53.0.CO;2-S 10542355

[bib46] Raineteau O Schwab ME 2001 Plasticity of motor systems after incomplete spinal cord injury. Nature reviews Neuroscience 2:263–273. doi: 10.1038/35067570 11283749

[bib47] Rogers BP Gore JC 2008 Empirical comparison of sources of variation for FMRI connectivity analysis. PLOS ONE 3:e3708. doi: 10.1371/journal.pone.0003708 19002252 PMC2577732

[bib48] Ruscheweyh R Sandkühler J 2002 Lamina-specific membrane and discharge properties of rat spinal dorsal horn neurones in vitro. The Journal of Physiology 541:231–244. doi: 10.1113/jphysiol.2002.017756 12015432 PMC2290304

[bib49] Smith SM 2012 The future of FMRI connectivity. Neuroimage 62:1257–1266. doi: 10.1016/j.neuroimage.2012.01.022 22248579

[bib50] Sprenger C Eippert F Finsterbusch J Bingel U Rose M Büchel C 2012 Attention modulates spinal cord responses to pain. Current Biology 22:1019–1022. doi: 10.1016/j.cub.2012.04.006 22608507

[bib51] Stroman PW Nance PW Ryner LN 1999 BOLD MRI of the human cervical spinal cord at 3 Tesla. Magnetic Resonance in Medicine 42:571–576. doi: 10.1002/(SICI)1522-2594(199909)42:33.0.CO;2-N 10467302

[bib52] Stroman PW Tomanek B Krause V Frankenstein UN Malisza KL 2002 Mapping of neuronal function in the healthy and injured spinal cord with spinal fMRI. Neuroimage 17:1854–1860 12498759 10.1006/nimg.2002.1305

[bib53] Stroman PW Kornelsen J Bergman A Krause V Ethans K Malisza KL Tomanek B 2004 Noninvasive assessment of the injured human spinal cord by means of functional magnetic resonance imaging. Spinal Cord 42:59–66. doi: 10.1038/sj.sc.3101559 14765137

[bib54] Summers PE Ferraro D Duzzi D Lui F Iannetti GD Porro CA 2010 A quantitative comparison of BOLD fMRI responses to noxious and innocuous stimuli in the human spinal cord. Neuroimage 50:1408–1415. doi: 10.1016/j.neuroimage.2010.01.043 20096788

[bib55] Valsasina P Agosta F Absinta M Sala S Caputo D Filippi M 2010 Cervical cord functional MRI changes in relapse-onset MS patients. Journal of neurology, neurosurgery, and psychiatry 81:405–408. doi: 10.1136/jnnp.2009.187526 19965858

[bib56] Valsasina P Rocca MA Absinta M Agosta F Caputo D Comi G Filippi M 2012 Cervical cord FMRI abnormalities differ between the progressive forms of multiple sclerosis. Human Brain Mapping 33:2072–2080. doi: 10.1002/hbm.21346 22887824 PMC6870085

[bib57] van den Heuvel MP Sporns O 2013 An anatomical substrate for integration among functional networks in human cortex. The Journal of Neuroscience: the Official Journal of the Society for Neuroscience 33:14489–14500. doi: 10.1523/JNEUROSCI.2128-13.2013 24005300 PMC6618386

[bib58] van der Meulen P Groen JP Tinus AM Bruntink G 1988 Fast field echo imaging: an overview and contrast calculations. Magnetic Resonance Imaging 6:355–368 3054380 10.1016/0730-725x(88)90472-9

[bib59] Wei P Li J Gao F Ye D Zhong Q Liu S 2010 Resting state networks in human cervical spinal cord observed with fMRI. European Journal of Applied Physiology 108:265–271. doi: 10.1007/s00421-009-1205-4 19777254

[bib60] Xie G Piché M Khoshnejad M Perlbarg V Chen JI Hoge RD Cohen-Adad J 2012 Reduction of physiological noise with independent component analysis improves the detection of nociceptive responses with fMRI of the human spinal cord. Neuroimage 63:245–252. doi: 10.1016/j.neuroimage.2012.06.057 22776463

[bib61] Yoshizawa T Nose T Moore GJ Sillerud LO 1996 Functional magnetic resonance imaging of motor activation in the human cervical spinal cord. Neuroimage 4:174–182. doi: 10.1006/nimg.1996.0068 9345507

